# Impact of Registered Dietitian Expertise in Health Guidance for Weight Loss

**DOI:** 10.1371/journal.pone.0151456

**Published:** 2016-03-22

**Authors:** Mie Imanaka, Masahiko Ando, Tetsuhisa Kitamura, Takashi Kawamura

**Affiliations:** 1 Department of Health and Nutrition, Kyoto Koka Women’s University, Kyoto, Japan; 2 Center for Advanced Medicine and Clinical Research, Nagoya University Hospital, Nagoya, Japan; 3 Division of Environmental Medicine and Population Sciences, Department of Social and Environmental Medicine, Graduate School of Medicine, Osaka University, Suita, Osaka, Japan; 4 Kyoto University Health Service, Kyoto, Japan; Central Queensland University, AUSTRALIA

## Abstract

**Background & Objectives:**

Expertise of registered dietitians (RDs) is important for health guidance but has been poorly evaluated. We evaluated the kind of RD expertise that would improve their skills.

**Design, Setting, Participants, Measurements:**

This study was a post-hoc analysis of our randomized controlled trial, which compared the weight change between participants using the web-based self-disclosure health support and those using the email health support. Healthy men and women aged 35–64 years with a body mass index (BMI) of > = 24.5 kg/m^2^ were recruited for this study. We evaluated the relationship of RD expertise indicators including the duration of working as an RD, the experience of health counseling, and membership in the Japan Dietetic Association (JDA) with the weight loss of study participants. The primary endpoint was the change in body weight. Comparison of changes in body weight by the RD expertise indicators was evaluated using analysis of covariance.

**Results:**

A total of 175 participants were eligible for analyses. Changes in body weight were significantly greater when they were supported by the RDs in the routine counseling group than when supported by the RDs in the non-routine counseling group (-1.8 kg versus -0.4 kg, fully adjusted P = 0.0089). Duration of working as an RD and JDA membership did not significantly affect changes in body weight.

**Conclusions:**

Among some indices of RD experience, the experience of providing routine experience of health counseling was associated with weight loss.

## Introduction

Obesity is one of the major public health problems, which may lead to cardiovascular or non-communicable diseases such as diabetes in advanced countries [1]. Weight management is an important approach to prevent such diseases [2]. Although it is difficult for obese persons to change their diet behavior by themselves, behavioral support by a registered dietitian (RD) is deemed to be effective to reduce obesity [3–5]. RD is a nationally certified category of specialized occupations for the provision of nutritional guidance to prevent diseases.

In health support for weight loss, individual face-to-face counseling considering one’s biomedical background and personal characteristics and small-group workshops using group dynamics have been generally carried out [6,7]. These days, information technology such as world-wide web, and person-to-person e-mail has been used in nutritional counseling [8–10]. The skill of RDs, therefore, would be required to conduct health support in various situations [11–12]. Although it was noted that guidance on weight management should be provided by experienced advisors in previous studies, detailed indices of such experience have not been specified.

Recently, we developed a web-based self-disclosure health support (WSHS) system for weight loss. In our randomized trial, we compared the weight change between the WSHS system and the e-mail health support (EHS) system and found that the WSHS system would be significantly more effective than the EHS system for short-term weight loss [13]. Using this dataset, we evaluated the relationship between the expertise of the supporting RDs and weight loss of the study participants.

## Methods

### Study design

This study was a post-hoc analysis of our randomized controlled trial (Clinical Trial Registration Number: UMIN000009147). All procedures complied with the Declaration of Helsinki and were approved by the Ethics Committee of Kyoto University Graduate School of Medicine.

### Participants and interventions

The newly developed a WSHS system using forced self-disclosure was significantly more effective than the EMS system for short-term weight loss. Details of the study were previously described [13]. This study was carried out from July 2008 to February 2009. Apparently healthy men and women aged 35–64 years with a body mass index (BMI) of > = 24.5 kg/m^2^ in their latest health examination were invited to an initial face-to-face guidance session and those who submitted written informed consent were enrolled in this study. Those who had been receiving dietary and exercise therapies, or who could not access Internet, or who had a current BMI <24.5 kg/m^2^ were excluded from our study. During the first interview, baseline data regarding the sex, age, height and weight, and waist circumference were collected from all subjects by the same dietitian. The weight and waist circumference were also measured by that dietitian 12 weeks after health support.

Participants were randomly assigned to either the WSHS group or the EHS group using the minimization method, balancing gender, age, and BMI, and then were assigned to one of the counselor-dietitians. A total of 13 RDs provided nutritional counseling under the direction of the principal dietitian. Each counselor-dietitian was allocated to both one of the WSHS groups and one of the EHS groups with the respective 6–8 participants in order to minimize the inter-group differences in dietitians' counseling. Furthermore, to ensure the homogeneity of nutritional guidance among the RD, advice for members of both groups was provided, based on a standardized manual [14] offered by the principal dietitian.

The WSHS group participants could receive advice from the corresponding dietitian and view other participant’s progress when they accessed this web system, while the EHS group participants could only receive advice from the corresponding dietitian by email. The follow-up period was 12 weeks for both groups.

### Key expertise indicator definition

This study obtained the following three RD expertise indicators: duration of work as an RD, experience of health counseling, and membership in the Japan Dietetic Association (JDA). In the present study, the number of years of working as an RD was focused on, and it was used as an evaluation criterion to define experienced advisors. The presence/absence of experience of providing routine experience of health counseling was also used as an evaluation criterion based on the idea that such experience in actual settings influences the improvement of experienced advisors’ skills. Furthermore, considering that membership of the American Dietetic Association (ADA) is regarded as an index of RD skills in the United States, being a member of the JDA was also used as an evaluation criterion in the present study. Duration of work as an RD was the number of years from the registration of RD to the participation in this study and the period of not working as an RD was excluded. This duration was stratified in 5-year increments. Experience of health counseling as an RD was obtained in our interview or her resume. Health counseling was defined as at least 20-minute individual counseling or at least 80-minute group counseling, both for lifestyle modification [15]. In this study, we classified experience of health counseling into two groups by frequency: once and more per week (routine) or less than once per week (non-routine) counseling. Many RDs and RD training institutions were affiliated with JDA, which has been contributing to the improvement of people’s nutrition through various kinds of activities [16]. We referred to the JDA membership list for the study.

### Outcome measures

The primary endpoint was the change in body weight of the participants in the health guidance. The secondary endpoint was the change in their waist circumference.

### Statistical analysis

Summary statistics were expressed by mean ± standard deviation (SD) for numerical variables and percentages for categorical variables. Changes in body weight and waist circumference by the RD’s expertise indicators were compared using analysis of covariance adjusting for gender (men/women), age (continuous values), and the assigned group (WSHS/EHS) at the health guidance. When evaluating one expertise indicator, other indicators were treated as adjusting factors. All statistical analyses were performed using JMP® 9 statistical software (SAS Institute, Inc., Cary, NC.). All tests were 2-tailed, and P values of <0.05 were considered statistically significant.

## Results

### Participant characteristics

A total of 196 participants were enrolled in this trial. Excluding three participants with a BMI of <24.5 at the first guidance session, the remaining 193 participants received the subsequent health counseling for weight loss. Eighteen participants were censored during the study period, and 175 (90.7%) were eligible for our analysis.

Characteristics of the RDs and the participants of the study are shown in Tables 1 and 2, respectively. RDs were all women and mean age was 32.2 years. Mean duration of work as an RD was 8.0 years. The proportion of RDs who had experience in routine health counseling and JDA membership was 53.8% and 46.2%, respectively. As for study participants, male patients accounted for 86.9% and mean age was 50.2 years. Changes in body weight and waist circumstance were -1.1 kg and -3.2 cm, respectively. The period of guidance provided by the 7 RD with experience of providing daily health guidance ranged from 2 to 14 (median: 4) years, confirming that all these RD had experience of providing such guidance for 2 years or longer. Similarly, the duration of JAD membership among the 6 members ranged from 4 to 18 (median: 10) years, indicating that all these members had maintained such membership for 4 years or longer.

**Table 1:** Characteristics of supporting registered dietitians.

Women, n (%)	13 (100.0)
Age, year, mean ± SD	32.2 ± 10.0
Duration of working as a registered dietitian, year, mean ± SD	8.0 ± 6.8
Routine experience of health counseling, n (%)	7 (53.8)
Membership of the Japan Dietetic Association, n (%)	6 (46.2)

SD, standard deviation.

**Table 2:** Characteristics of study participants.

	At first guidance	After health support	Changes in outcomes
Men, n (%)	152 (86.9)		
Age, year, mean ± SD	50.2 ± 7.4		
Body weight, kg, mean ± SD	78.0 ± 10.4	76.9 ± 10.6	-1.1 ± 2.6
Waist circumference, cm, mean ± SD*	94.4 ± 7.4	91.4 ± 7.7	-3.2 ± 3.6

SD, standard deviation.

*Data were obtained from 163 participants.

### RD's expertise indicators and changes in body weight of participants

Table 3 shows the relationship between RD expertise indicators and changes in body weight of the participants. Body weight was significantly more reduced when supported by RDs in the routine counseling group than when supported by RDs in the non-routine counseling group (-1.8 kg versus -0.4 kg, fully adjusted P = 0.0089). As for duration of work as an RD and JDA membership, participants’ body weight reduction was significantly greater for long-working RDs and JDA members, but the significance of changes in body weight disappeared after full adjustment. Changes in waist circumstance by RD expertise indicators were similar to those in body weight, but all of the test results were statistically insignificant (Table 4).

**Table 3:** Relationship between registered dietitian's expertise indicators and changes in body weight of participants.

	Registered dietitian	Participants	Changes in weight	*P* values*		
Factors	N (%)	N (%)	(mean ± SD)	Unadjusted	Adjusted 1^†^	Adjusted 2^‡^
Duration of working as a registered dietitian				0.0013	0.0035	0.64
1–5 years	7 (53.8)	96 (54.9)	-0.5 ± 2.1			
5–10 years	3 (23.1)	39 (22.3)	-1.8 ± 3.0			
> = 10 years	3 (23.1)	40 (22.8)	-1.9 ± 2.9			
Experience of health counseling				0.0003	0.0001	0.0089
Routine	7 (53.8)	91 (52.0)	-1.8 ± 2.6			
Non-routine	6 (46.2)	84 (48.0)	-0.4 ±2.4			
Membership of the Japan Dietetic Association				0.0006	0.002	0.33
Member	6 (46.2)	79 (45.1)	-1.8 ± 2.9			
Non-member	7 (53.8)	96 (54.9)	-0.5 ± 2.1			

*Analysis of covariance.

^†^*P* values were adjusted for participants' gender, age, and the assigned group at the first guidance.

^‡^*P* values were adjusted for participants' gender, age, and the assigned group at the first guidance, and dietitian's expertise indicators.

SD, standard deviation.

**Table 4:** Relationship between registered dietitian's expertise indicators and changes in waist circumference of participants.

	Registered dietitian	Participants	Changes in waist	*P* values*		
Factors	N (%)	N (%)	(mean ± SD)	Unadjusted	Adjusted 1^†^	Adjusted 2^‡^
Duration of working as a registered dietitian				0.24	0.30	0.67
1–5 years	7 (53.8)	89 (54.6)	-2.9 ± 3.2			
5–10 years	3 (23.1)	38 (23.3)	-3.6 ±4.3			
> = 10 years	3 (23.1)	36 (22.1)	-3.6 ± 3.8			
Experience of health counseling				0.06	0.04	0.07
Routine	6 (46.2)	85 (52.1)	-3.7 ± 3.6			
Non-routine	7 (53.8)	78 (47.9)	-2.7 ± 3.5			
Membership of the Japan Dietetic Association				0.19	0.27	0.74
Member	6 (46.2)	74 (45.4)	-3.6 ± 4.0			
Non-member	7 (53.8)	89 (54.6)	-2.9 ± 3.2			

*Analysis of covariance.

^†^*P* values were adjusted for participants' gender, age, and the assigned group at the first guidance.

^‡^*P* values were adjusted for participants' gender, age, and the assigned group at the first guidance, and dietitian's expertise indicators.

SD, standard deviation.

## Discussion

In this sub-study of our randomized controlled trial, we evaluated the relationship between RD expertise indicators and the magnitude of weight loss of the study participants. Here, we found that RD experience of routine health counseling was significantly associated with participants’ weight loss.

It was noted that guidance should be provided by experienced advisors in previous studies. For example, previous studies showed that RD experience was important in health support including glycemic control and weight loss [5,11] and experience-based skills for the face-to-face counseling would result in substantial weight loss [11]. Communication has been reported to be important for the provision of face-to-face guidance and health support. Recently, training in professional skills is required in various occupations. For example, objective structured clinical examination using simulated patients was introduced to medical education to improve clinical practice of medical students [17]. The necessity of experience of community-based activities has also been noted in the field of public health education. One report mentioned that the accumulation of experience was needed to revitalize primary care social service [18]. Similarly, RDs should be also routinely engaged in health support in healthcare centers or welfare facilities in order to perform effective weight loss counseling. The results of the present study indicate that the presence/absence of experience of providing daily guidance in actual settings is a key evaluation criterion.

In this study, duration of work as an RD did not affect the changes in participants’ body weight. The influence of years of working in clinical practice is still under debate. The number of years of working as an RD was used as an index of experienced advisors in the present study. Such a number has been reported to be important to define experienced individuals [19]. For example, in the field of dentistry, the length of professional experience would contribute to work efficiency in dental practice [20]. Nurses having longer clinical experience could also give better guidance to the patients considering their individuality and independence [21]. On the other hand, when one’s experience exceeded a certain level, its incremental effects would be attenuated [22]. RDs have various kinds of missions including cooking, menu planning, counseling, and education [23,24]. Thus, other vocations than counseling might contribute less to the improvement of counseling skills.

Our study underscored that JDA membership was not associated with changes in participants’ body weight. However, in the United States, membership in the American Dietetic Association was one of the determining factors for evaluation of RD capability [25]. The JDA has continuously offered various kinds of seminars through which many RDs have acquired skills and knowledge. Considering the importance of the experience of health counseling shown in this study, we propose that the JDA should also offer more practical training courses in collaboration with patient societies and health promotion facilities.

This study also evaluated the reduction in waist circumference. We found similar relationships between RD expertise indicators and participants’ waist circumference changes but the association did not reach statistical significance. It is known that errors in waist circumference measurement are marked [26]. And this might cause the unstableness of waist circumference measurements.

This study has some inherent limitations. First, this was a post-hoc analysis of our preceding randomized controlled study, and the number of measured expertise indicators was limited. This is a secondary study on RCT, and the results are not sufficient to comprehensively and systematically evaluate advisors’ skills, indicating the necessity of more detailed studies to confirm them. Second, this study evaluated changes in weight and waist circumference. The true end point of health support should be a reduction in mortality and morbidity from critical diseases. However, weight change could be a reasonable short-term surrogate marker because weight loss could prevent cardiovascular events [27]. The shortness of our observation is another limitation because long-term changes or fluctuation in body weight are unknown. When evaluating experience, it may be important to consider not only its period, but also its quality.

In this study assessing the relationship between RD expertise indicators and the magnitude of weight loss, RD routine health counseling experience served to significantly enhance weight loss of overweight persons. RDs should therefore accumulate greater experience in health counseling in the real word setting in order to effectively reduce the type of obesity prevailing in industrialized countries. As future perspectives, further studies will be conducted to examine sociodemographic characteristics, education, training, and specialized experience, as well as the type of employment, as more appropriate evaluation criteria.
